# Receptor-Interacting Protein Kinase 3 Inhibition Relieves Mechanical Allodynia and Suppresses NLRP3 Inflammasome and NF-κB in a Rat Model of Spinal Cord Injury

**DOI:** 10.3389/fnmol.2022.861312

**Published:** 2022-04-19

**Authors:** Song Xue, Zhen-xin Cao, Jun-nan Wang, Qing-xiang Zhao, Jie Han, Wen-jie Yang, Tao Sun

**Affiliations:** ^1^Department of Pain Management, Shandong Provincial Hospital, Cheeloo College of Medicine, Shandong University, Jinan, China; ^2^Departments of Pain Management, Shandong Provincial Hospital Affiliated to Shandong First Medical University, Jinan, China

**Keywords:** neuropathic pain after spinal cord injury, receptor-interacting protein kinase 3, NLRP3 inflammasome, nuclear factor-kappa B (NF-κB), GSK872

## Abstract

**Background:**

Neuroinflammation is critical in developing and maintaining neuropathic pain after spinal cord injury (SCI). The receptor-interacting protein kinase 3 (RIPK3) has been shown to promote inflammatory response by exerting its non-necroptotic functions. In this study, we explored the involvement of RIPK3 in neuropathic pain after SCI.

**Methods:**

Thoracic (T10) SCI rat model was conducted, and the mechanical threshold in rats was measured. The expressions of RIPK3, nod-like receptor family pyrin domain-containing protein 3 (NLRP3), caspase-1, and nuclear factor-κB (NF-κB) were measured with western blotting analysis or quantitative real-time polymerase chain reaction (qRT-PCR). Double immunofluorescence staining was used to explore the colabeled NLRP3 with NeuN, glial fibrillary acidic protein (GFAP), and ionized calcium-binding adapter molecule 1 (IBA1). In addition, enzyme-linked immunosorbent assay (ELISA) was applied to analyze the levels of proinflammatory factors interleukin 1 beta (IL-1β), interleukin 18 (IL-18), and tumor necrosis factor alpha (TNF-α).

**Results:**

The expression of RIPK3 was elevated from postoperative days 7–21, which was consistent with the development of mechanical allodynia. Intrathecal administration of RIPK3 inhibitor GSK872 could alleviate the mechanical allodynia in SCI rats and reduce the expression levels of RIPK3. The activation of NLRP3 inflammasome and NF-κB was attenuated by GSK872 treatment. Furthermore, immunofluorescence suggested that NLRP3 had colocalization with glial cells and neurons in the L4–L6 spinal dorsal horns. In addition, GSK872 treatment reduced the production of inflammatory cytokines.

**Conclusion:**

Our findings indicated that RIPK3 was an important facilitated factor for SCI-induced mechanical allodynia. RIPK3 inhibition might relieve mechanical allodynia by inhibiting NLRP3 inflammasome, NF-κB, and the associated inflammation.

## Introduction

Neuropathic pain is a common complication after spinal cord injury (SCI) (Bryce et al., 2012; Sweis and Biller, 2017). The neuropathic pain after SCI is stubborn, severe, and protracted, which brings great pain and endless torture to the patients (Attal, 2021). The sense of helplessness and despair that pain brings to patients is even more harmful than the impact of dysfunction. Unfortunately, there is still a lack of effective treatment to control the progression of pain. Therefore, it is essential to explore the mechanisms of neuropathic pain after SCI to develop effective treatment programs or drugs.

The pathophysiological mechanism of neuropathic pain after SCI is complex. However, the previous research suggested that neuroinflammation in the remote spinal dorsal horn might play an especially critical role in the development course of neuropathic pain after SCI (Gwak and Hulsebosch, 2009). It was confirmed that activated glial cells and inflammatory factors in the remote spinal dorsal horns promoted the induction and maintenance of neuropathic pain after SCI by increasing neurons' excitability (Detloff et al., 2008; Sandhir et al., 2011; Gwak et al., 2012). However, the upstream kinases or signal pathways that promote neuroinflammation in remote spinal dorsal horns have not been well-studied.

Receptor-interacting protein kinase 3 (RIPK3) is a crucial threonine–serine protein kinase for necroptosis (Khan et al., 2014). Tumor necrosis factors, toll-like receptor agonists, oxidative stress, and virus infection could activate RIPK3 and trigger necroptosis (Kaczmarek et al., 2013). It is well-known that necroptosis is a highly inflammatory cell death that can trigger inflammatory responses due to the release of intracellular immunogenic contents. Interestingly, the recent studies demonstrate that activated RIPK3 may contribute to the inflammation by exerting its non-necroptotic functions, such as activating nuclear factor-κB (NF-κB) or nod-like receptor family pyrin domain-containing protein 3 (NLRP3) inflammasome (Moriwaki et al., 2014) (Moriwaki and Chan, 2017). Both NF-κB and NLRP3 inflammasome are the important pathways for promoting inflammatory. NF-κB is a transcriptional activator of inflammatory factor genes (Lawrence, 2009). NLRP3 inflammasome is a protein complex composed of NLRP3, the adaptor apoptosis-associated speck-like protein containing a CARD (ASC) and caspase-1 (Kelley et al., 2019). When stimulated by the endogenous and exogenous dangerous signals, the assembled NLRP3 inflammasome activates caspase-1, which converts the pro-IL-1β and pro-IL-18 into mature interleukin 1 beta (IL-1β) and interleukin 18 (IL-18) (Kelley et al., 2019).

The previous studies showed that RIPK3 might be involved in inflammatory bowel disease (Lee et al., 2020) and autoinflammatory disease (Speir and Lawlor, 2021). RIPK3 has also been studied in the rodent SCI model. A previous study showed that RIPK3 was highly expressed at the injured site for 21 days after SCI and localized in neurons, astrocytes, and oligodendrocytes (Kanno et al., 2015). Furthermore, the recent investigations have found that inhibiting RIPK3-mediated necroptosis helped to reduce neuroinflammation and the recovery of locomotion (Wang et al., 2019; Hongna et al., 2021). Nevertheless, a few studies investigated the expression of RIPK3 in the remote spinal cord after SCI, and whether RIPK3 inhibition could relieve neuropathic pain after SCI was also unclear.

To solve these problems, by establishing the thoracic SCI model, we studied the RIPK3 expression in the remote spinal dorsal horns and further explored its role in neuropathic pain after SCI. Furthermore, we examined the effect of RIPK3 inhibitor GSK872 on the expression of the NLRP3 inflammasome, NF-κB, and proinflammatory cytokines.

## Materials and Methods

### Animals and Grouping

This experimental object was the male SD rats (6–7 weeks, 210–260 g) provided by the Shandong University Laboratory. All experimental rats were fed with a 12-h light/dark cycle at 25°C and had free access to rodent water and food. The Animal Care and Use Committee at Shandong University approved our experimental designs and operation procedures.

All rats were randomly divided into four groups (*n* = 4–7 per group for various analyses): sham group, SCI group, vehicle group, GSK872 group. In the sham group, only the vertebral lamina was removed without SCI; In the vehicle group, rats with SCI received an intrathecal injection of 10% dimethyl sulfoxide (DMSO) (Cell Signaling Technology, USA); In the GSK872 group, rats with SCI received an intrathecal injection of GSK872 (Med Chem Express, China). GSK872 was dissolved in DMSO.

### SCI Model

Spinal cord injury model was made using modified Allen's method (Khan et al., 1999). Rats were anesthetized by intraperitoneal injection of 30 mg/kg of pentobarbital. The skin around the T10 segment in the back was disinfected and a longitudinal incision was made. The tendons and muscle tissue were separated to expose the T10 spinous processes and lamina, and then, the T10 lamina was removed, which exposes the corresponding spinal cord. A 10-g iron bar was used to cause SCI, vertically dropped from a height of 30 mm through a glass tube onto the exposed spinal cord. The hemostatic suture was performed using 3-0 silk thread, and antibiotics were then injected subcutaneously. Only the vertebral lamina was removed in the sham group without SCI. Rats were intramuscularly injected with 20 × 10^4^ U/d penicillin for 5 days and received artificial micturition two times a day until the recovery of micturition function.

### Intrathecal Injection and Drug Administration

The direct transcutaneous intrathecal injection was based on the method reported by Mestre et al. (1994). In brief, rats were anesthetized by inhaling enflurane, and then, the hip tubercle was touched to determine the L5 or L6. The 25-μl microinjection syringe (Shanghai Gaoge Industry and Trade Co., Ltd) was inserted into L5 or L6 intervertebral space vertically until the occurrence of tail-flick reflex, which indicated the tip of the needle had reached the subarachnoid space. When the tail-flick reflex was observed, the needle insertion was stopped and injected 10 μl GSK872 (25 mmol/L) (Hou et al., 2018) or 10 μl of DMSO. Rats were injected with GSK872 or DMSO 30 min before the surgery, 1 day, and 2 days after the surgery.

### Pain Behavior Assessment

A total of 50% paw withdrawal threshold (PWT) was used to assess the mechanical allodynia according to the previous reports (Chaplan et al., 1994). Observers were blinded to the experimental groups and recorded 50% PWT on the first day before surgery and on the 7th, 10th, 14th, 17th, and 21st days after the operation. Before each measurement, it was necessary to let the rats adapt to the watch box for at least 30 min until exploration activities disappeared.

A total of eight von Frey hairs (0.4, 0.6, 1, 2, 4, 6, 8, and 15.0 g, Stoelting, United States) were used to measure 50% PWT in rat hind paws according to the “Up-Down” method. The filament of 2 g was used first. Then, the intensity of the next filament was decreased when the animal reacted or increased when the animal did not respond. Withdrawal of claws, shaking or licking was considered a painful reaction. When the response change was observed for the first time, this procedure was continued for six stimuli. Fifty percentage PWT was calculated using the following formula: 50% PWT = 10^(Xf+κδ)^ (Xf is the logarithm value of the last von Frey fiber, and *K* is the corresponding value of the sequence, δ = 0.224). Bilateral rat hind paws were tested. Finally, the average of 50% PWT of bilateral hind paws was taken.

### Tissue Sample Collection

To explore the protein and mRNA expression of RIPK3 at different time points after the operation, the rats in the SCI group were sacrificed by pentobarbital anesthesia (60 mg/kg, i.p) at postoperative days 7, 14, and 21 after conducting the pain behavior assessment. Rats in the sham group were euthanized on postoperative day 21. To study the effect of GSK872 on the expression of target molecules, all rats in each group (sham, SCI, vehicle, and GSK872 groups) were sacrificed at postoperative day 7. In double immunofluorescence staining, the rats were cardiac infused with 0.9% NaCl and 4% paraformaldehyde, and then, the L4–L6 spinal cord was separated from the rats and fixed with 4% paraformaldehyde. For other molecular detection, bilateral spinal dorsal horns (L4–L6) were collected, frozen in liquid nitrogen, and stored at −80°C until further analysis.

### Western Blotting

Total proteins from tissues were extracted in RIPA lysis buffer (Solarbio, China), and a bicinchoninic acid (BCA) protein assay kit (Solarbio, China) was used for evaluating the protein concentration. The sample proteins were separated by sodium dodecyl sulfate–polyacrylamide gel electrophoresis and transferred onto polyvinylidene difluoride membranes. The membranes were blocked with 5% non-fat milk in TBST followed by incubating with primary antibody overnight. The primary antibodies were as follows: anti-RIPK3 (1:1,200; Novus Biologicals), anti-NLRP3 (1:400; Novus Biologicals), anti-caspase-1 (1:800; Novus Biologicals), anti- NF-κB/p65 (1:1,000; Cell Signaling Technology, CST), anti-β-actin (1:5,000; Proteintech Group, PTG), and anti-glyceraldehyde 3-phosphate dihydrogen (GAPDH) (1:5,000; Proteintech Group, PTG). After being washed with Tris Buffered Saline with Tween (TBST), the membranes were incubated with goat anti-rabbit second antibody for 1.5 h. Finally, the enhanced chemiluminescence (ECL; Thermo Scientific) was used to visualize immunoblots, and the densities of the relative target proteins were measured using ImageJ. The GAPDH or β-actin was chosen as the internal reference control.

### ELISA

On the 7th postoperative, the bilateral spinal dorsal horns were ground to tissue homogenization and were centrifuged at 10,000 rpm at 4°C for 30 min. After the supernatant was collected, we used enzyme-linked immunosorbent assay (ELISA) kits (TNF-α: Westang, China; IL-1β: MultiSciences, China; IL-18: Westang, China) to detect the levels of TNF-α, IL-1β, and IL-18 according to the instructions of ELISA kits.

### qRT-PCR

According to the instructions, total RNAs were extracted from the bilateral dorsal horns using RNAex Pro Reagent (AG21102, Accurate Biotechnology, Hunan, China). Complementary DNA (cDNA) was synthesized using Evo M-MLV RT Mix Kit (AG11728, Accurate Biotechnology, Hunan, China). Polymerase chain reaction (PCR) amplifications were conducted using SYBR® Green Premix Pro Taq HS qPCR kit (AG11701, Accurate Biotechnology, Hunan, China). Real-time fluorescent quantitative PCR was carried out using Light Cycler® 480 II (Roche, Switzerland). β-actin was served as the internal reference for normalization. The mRNA levels of RIPK3, NLRP3, and caspase-1 were calculated using the 2^−ΔΔCT^ method. The primers for NLRP3, RIPK3, caspase-1, and β-actin are shown in Table 1.

**Table 1:** Sequences of primers.

**Gene**	**Forward primer**	**Reverse primer**
RIPK3	CTACTGCACCGGGACCTCAA	GTGGACAGGCCAAAGT CTGCTA
NLRP3	CTGAAGCATCTGCAACC	AACCAATGCGAGATCCTG ACAAC
caspase-1	ACTCGTACACGTCTTGCC CTCA	CTGGGCAGGCAGCAAA TTC
β-actin	GGAGATTACTGCCCTGGCT CCTA	GACTCATCGTACTCCTG CTTGCTG

### Double Immunofluorescence Staining

On postoperative day 7, the L4–L6 spinal cord was harvested from rats (*n* = 3 for SCI group), embedded in paraffin, and cut into 20-μm-thick sections. These sections (*n* = 3 for each sample) were dewaxed, dehydrated by gradient alcohol, and repaired by antigen. Next, the sections were blocked with endogenous peroxidase and 10% donkey serum and then incubated with the following primary antibodies: NLRP3 (1:200; bs-6655R, Bioss), GFAP (1:500; GB12096, Servicebio), IBA-1(1:500; GB12105, Servicebio), and NeuN (1:100; GB13138-1, Servicebio) overnight at 4°C. After the primary antibody incubation, the sections were incubated with the corresponding secondary antibodies conjugated with CY3 and FITC for 1 h in dark conditions at 37°C. Finally, the dorsal horns were observed and photographed under a fluorescence microscope (Olympus, Japan).

### Statistical Analysis

SPSS 24.0 software (IBM, USA) was used to perform all the data analysis. All results were presented as mean ± SD. The Kolmogorov–Smirnov test was used to detect whether the data conformed to the normal distribution. For behavioral experiments, comparisons between multiple groups were conducted by repeated-measures analysis of variance and Tukey's *post-hoc* analysis. For quantitative real-time polymerase chain reaction (qRT-PCR), western blot, and ELISA, multiple-group comparisons were carried out by one-way ANOVA and Tukey's *post-hoc* analysis. When *p* < 0.05, we considered the difference between the two groups to be statistically significant.

## Results

### Upregulation of RIPK3 Expression in the SCI Model

**Figure 1:**
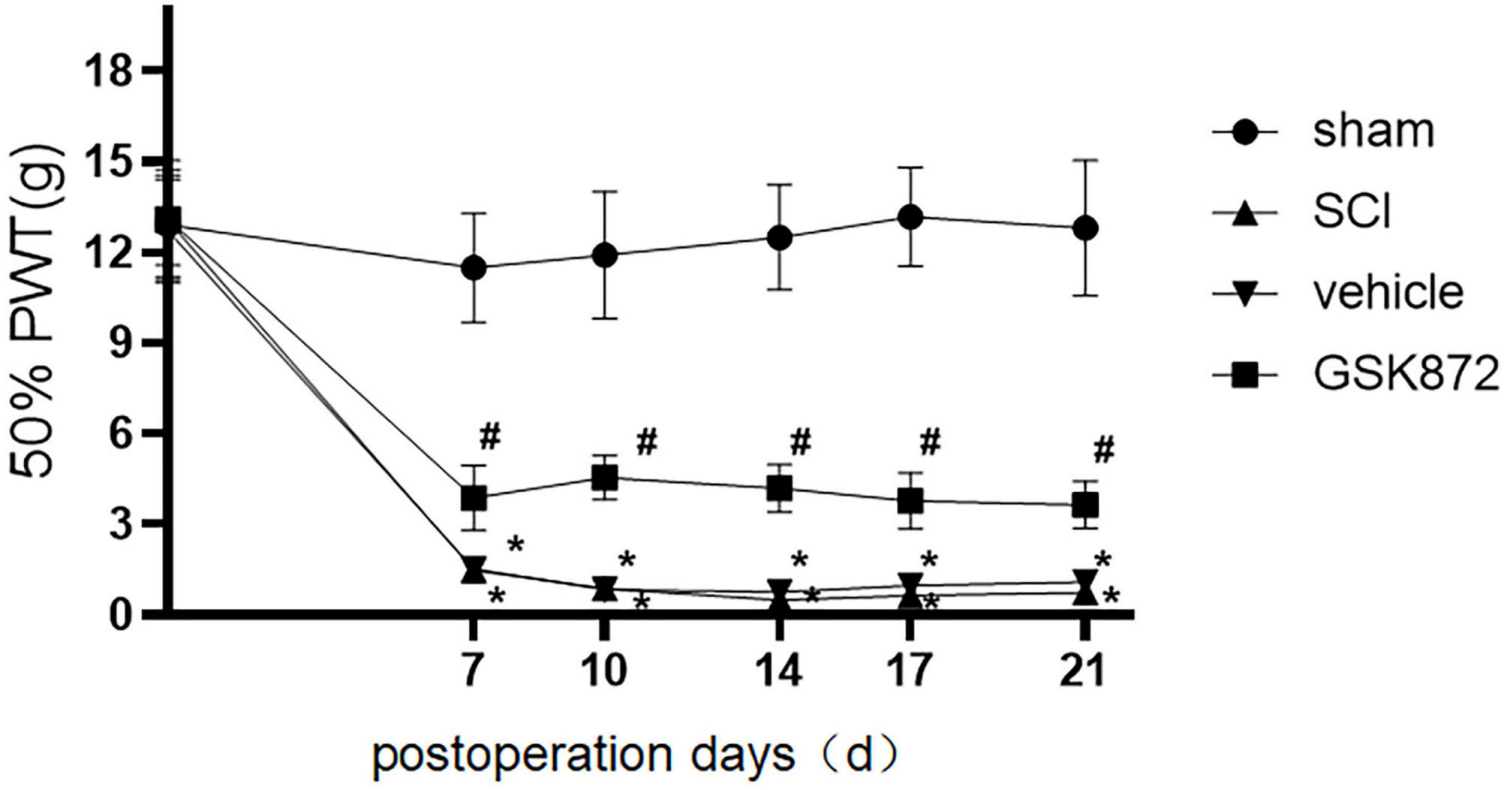
Intrathecal administration of GSK872 (10 μl, 25 mmol/L) attenuated mechanical hypersensitivity induced by SCI (*n* = 7 for each group). All data were calculated as mean ± SD. *p < 0.05 vs. the sham group; ^#^*p* < 0.05 the vehicle group. Comparisons between multiple groups were conducted by repeated-measures analysis of variance and Turkey's *post-hoc* analysis SCI: spinal cord injury. 50% PWT, fifty percentage paw withdrawal threshold.

Mechanical allodynia was tested using 50% PWT (Figure 1). Compared with the rats in sham group, rats in the SCI group developed mechanical allodynia from 7 to 21 days after the surgery (*p* < 0.05), which indicated that the SCI model was established successfully.

**Figure 2:**
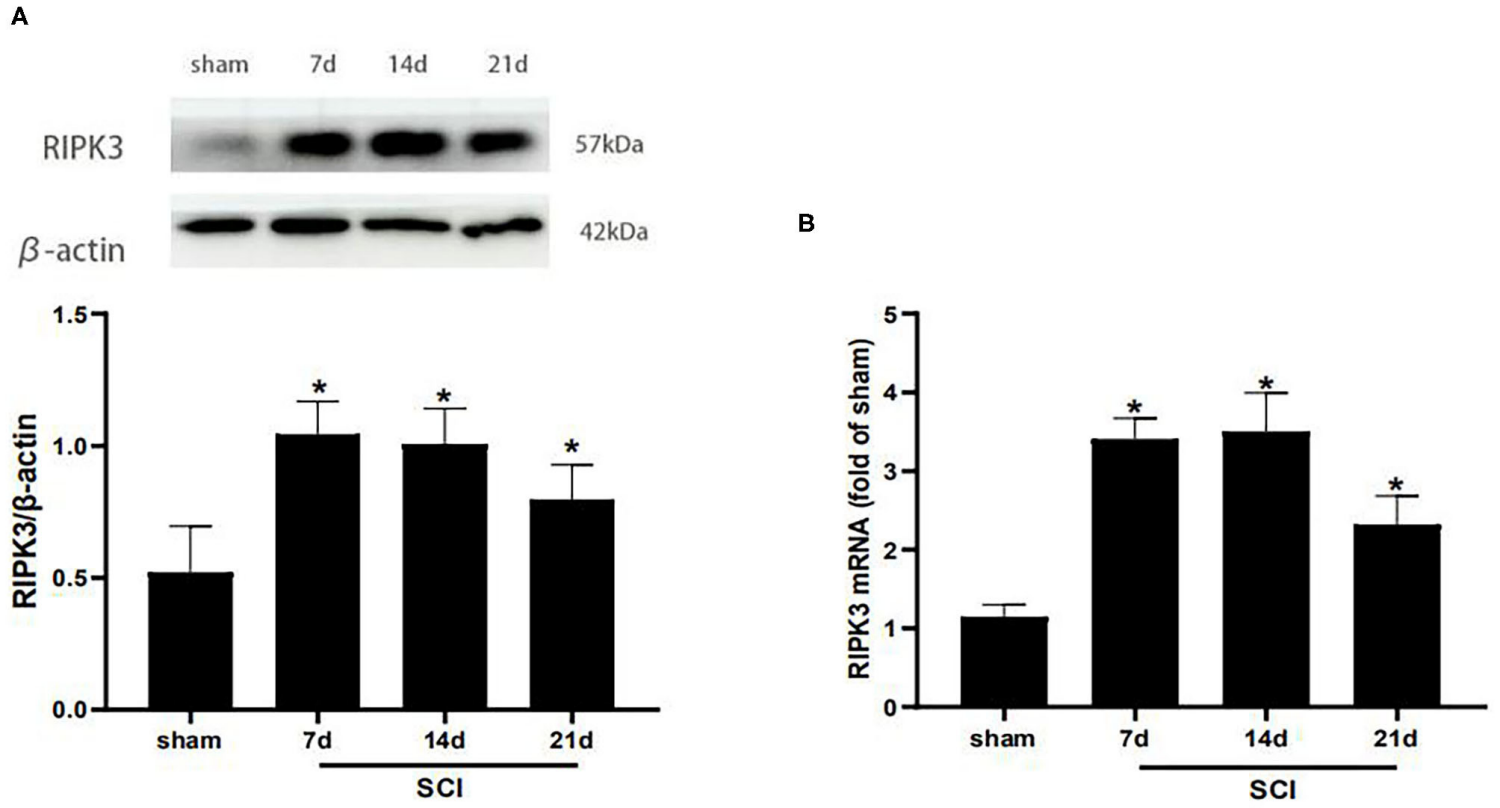
RIPK3 was up-regulated in the dorsal horns after thoracic SCI. **(A)** The expression of RIPK3 in the SCI group was measured by western blot analysis on the 7th, 14th, 21th postoperative days (*n* = 5 for each group). **(B)** Quantitative analysis of the mRNA expression of RIPK3 in the sham group and SCI group on the 7th, 14th, 21th postoperative days (*n* = 4 for each group). All data were calculated as mean ± SD. *p < 0.05, vs. the sham group. Comparisons between multiple groups were carried out by one-way ANOVA and Turkey's *post-hoc* analysis. RIPK3, receptor-interacting protein kinase 3.

Western blot analysis was carried out to detect the RIPK3 protein level at different time points postoperation (Figure 2A). Compared with the sham group, the protein expression of RIPK3 was significantly increased in SCI group from postoperative days 7–21 (*p* < 0.05). Meanwhile, similar results were verified using qRT-PCR (*p* < 0.05) (Figure 2B).

### GSK872 Decreased RIPK3 Expression Level and Relieved Mechanical Allodynia Induced by SCI

**Figure 3:**
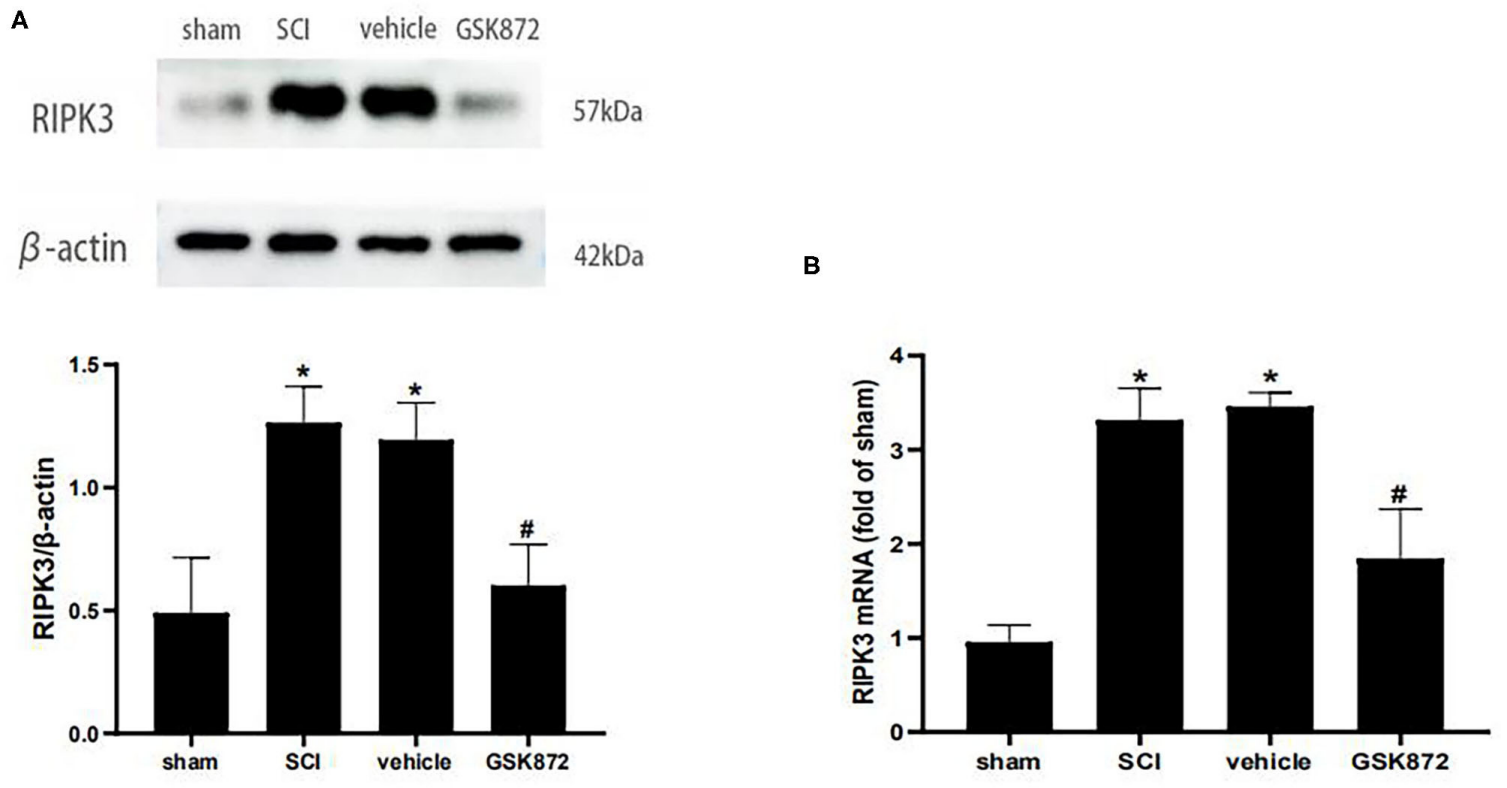
GSK872 (10 μl, 25 mmol/L) reduced the expression levels of RIPK3 after SCI. **(A)** The expression of RIPK3 in different groups, including sham, SCI, vehicle and GSK872 group, was measured by western blot analysis (*n* = 5 for each group). **(B)** Quantitative analysis of the RIPK3 mRNA expressions in different groups including sham, SCI, vehicle and GSK872 group (*n* = 4 for each group). All data were calculated as mean ± SD. *p < 0.05 vs. the sham group. ^#^*p* < 0.05 vs. the vehicle group. Comparisons between multiple groups were carried out by one-way ANOVA and Turkey's *post-hoc* analysis.

To determine whether RIPK3 was involved in neuropathic pain after SCI, the effect of GSK872 on mechanical allodynia and the RIPK3 expression level were assessed. About 10 μl GSK872(25 mmol/L) was injected 30 min before the surgery, 1 day, and 2 days after the surgery. Western blot analysis was performed to evaluate RIPK3 expression level (Figure 3A). The protein expression of RIPK3 was upregulated in vehicle rats compared with those in the sham group (*p* < 0.05). The upregulation of RIPK3 was wholly reversed after GSK872 treatment (*p* < 0.05). In addition, the results from qRT-PCR were consistent with the western blot analysis (*p* < 0.05) (Figure 3B).

As shown in Figure 1, 50% PWT was significantly decreased in the vehicle group compared with the sham group (*p* < 0.05). After intrathecal injection of RIPK3 inhibitor GSK872, mechanical allodynia in rats who received SCI was relieved from 7 to 21 days postoperation (*p* < 0.05).

### GSK872 Reduced the Production of Inflammatory Cytokines

**Figure 4:**
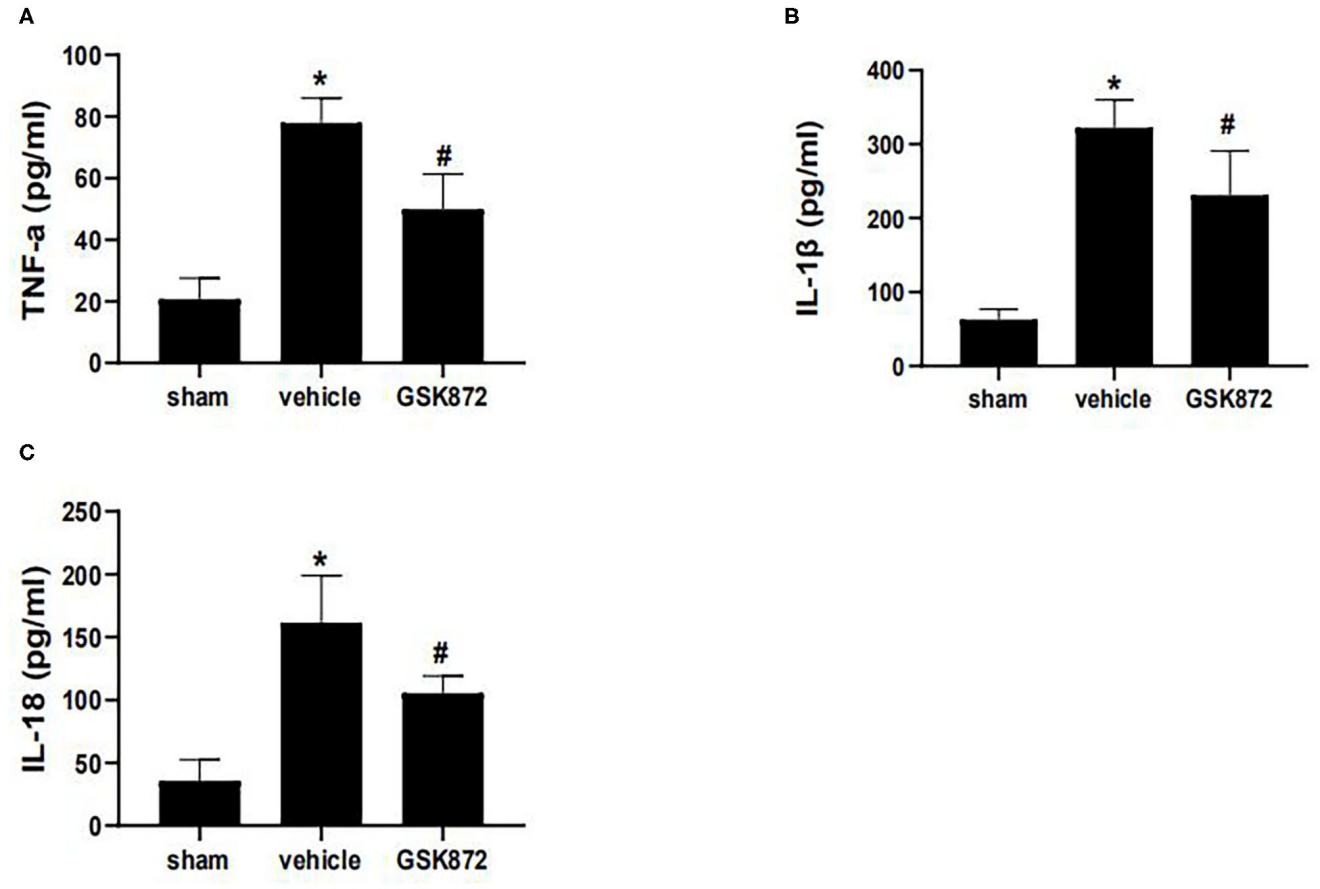
Intrathecal injection of GSK872 (10 μl, 25 mmol/L) inhibited neuroinflammation in the lumbar spinal dorsal horns (*n* = 4 for each group). **(A–C)** The levels of TNF-α, IL-1β, and IL-18 in different groups, including sham, vehicle and GSK872 group, were investigated by ELISA. The protein level of TNF-α, IL-1β, and IL-18 were reduced after GSK872 treatment. All data were calculated as mean ± SD. *p < 0.05 vs. the sham group. ^#^*p* < 0.05 vs. the vehicle group. Comparisons between multiple groups were carried out by one-way ANOVA and Turkey's *post-hoc* analysis. IL, interleukin; TNF-α, tumor necrosis factor-α.

To further investigate whether inhibition of RIPK3 could restrict neuroinflammation, ELISA was used to assess the levels of inflammatory cytokines (Figures 4A–C). The expressions of IL-1β (*p* < 0.05), IL-18 (*p* < 0.05), and TNF-α (*p* < 0.05) markedly ascended in the vehicle group compared with the sham group. The protein levels of TNF-α (*p* < 0.05), IL-1β (*p* < 0.05), and IL-18 (*p* < 0.05) were restricted in the GSK872 group compared with the vehicle group.

### GSK872 Suppressed the Activation of NLRP3 Inflammasome in Glia and Neurons

**Figure 5:**
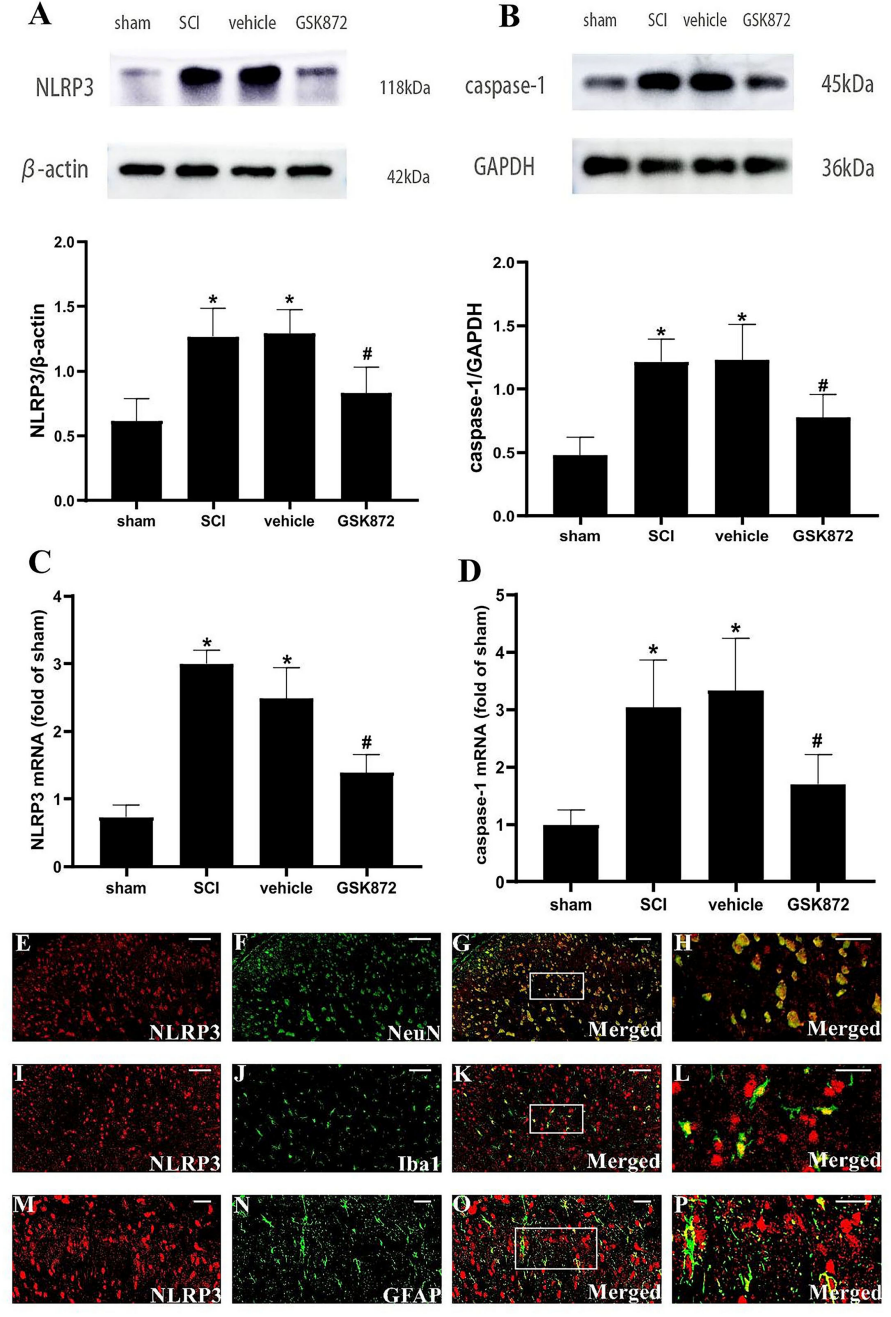
GSK872 (10 μl, 25 mmol/L) suppressed the activation of NLRP3 inflammasome in glia and neurons. **(A,B)** The expressions of NLRP3 and caspase-1 in different groups, including sham, SCI, vehicle and GSK872 group, were measured by western blot analysis (*n* = 5 for each group). **(C,D)** Quantitative analysis of NLRP3 and caspase-1 mRNA expressions in different groups including sham, SCI, vehicle and GSK872 group (*n* = 4 for each group). **(E–P)** Immunofluorescence staining of NLRP3 (red) with NeuN, a neuronal marker (green); GFAP, an astrocyte marker (green); and Iba-1, a microglial marker (green) in the lumbar dorsal horns of SCI rats. Scale bar, 50 μm **(E–G,I–K)**. Scale bar, 25 μm **(M–O)**. Scale bar, 20 μm **(H,L,P)**. All data were calculated as mean ± SD. *p < 0.05 vs. the sham group. ^#^*p* < 0.05 vs. the vehicle group. Comparisons between multiple groups were carried out by one-way ANOVA and Turkey's *post-hoc* analysis.

To further explore the potential molecular mechanism of GSK872 in alleviating mechanical allodynia, the expression of NLRP3 inflammasome was investigated. The results from western blot showed that the protein levels of NLRP3 (*p* < 0.05) and caspase-1 (*p* < 0.05) were remarkably upregulated in the vehicle group compared with the sham group (Figures 5A,B). Rats treated with GSK872 exhibited lower protein levels of NLRP3 (*p* < 0.05) and caspase-1 (*p* < 0.05) compared with the rats in the vehicle group. We also found similar results by examining the mRNA expression of NLRP3 (*p* < 0.05) and caspase-1 (*p* < 0.05) (Figures 5C,D).

In addition, the cellular localization of NLRP3 in nerve cells of spinal dorsal horns was researched (Figures 5E–P). Double immunofluorescent staining revealed that NLRP3 was localized in the microglia, neurons, and astrocytes in the dorsal horn of SCI rats.

### Effect of GSK872 on NF-κB p65

**Figure 6:**
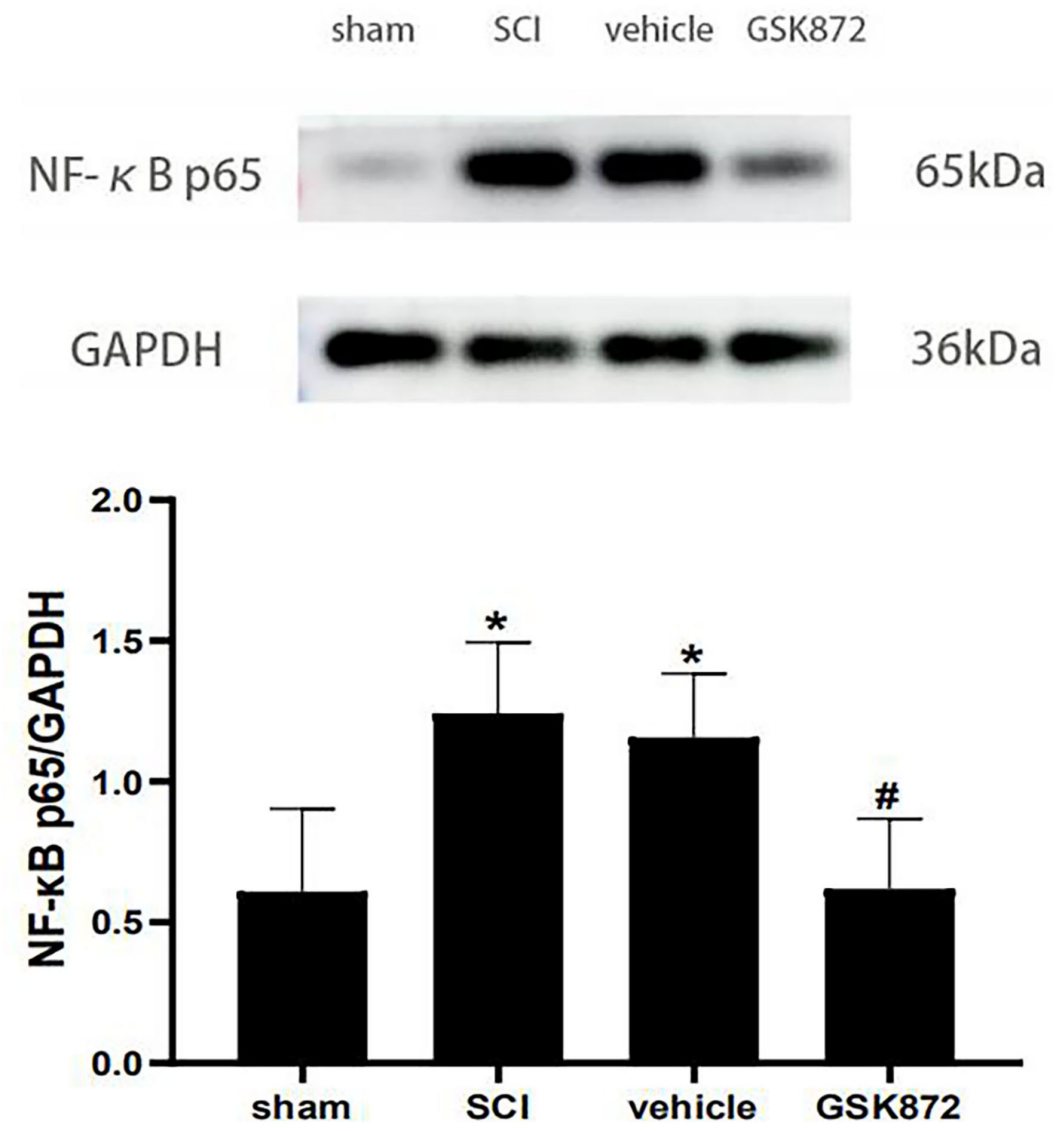
Intrathecal injection of GSK872 (10 μl, 25 mmol/L) inhibited NF-κB p65 in the lumbar spinal dorsal horns (*n* = 5 for each group). The expression of NF-κB p65 in different groups, including sham, SCI, vehicle and GSK872 group, was measured by western blot analysis. All data were calculated as mean ± SD. *p < 0.05 vs. the sham group. ^#^*p* < 0.05 vs. the vehicle group. Comparisons between multiple groups were carried out by one-way ANOVA and Turkey's *post-hoc* analysis. NF-κB, nuclear factor-kappa B.

The western blot was conducted to evaluate the expression level of NF-κB p65 (Figure 6). Our data showed that NF-κB p65 was markedly upregulated in the vehicle group compared with the sham group (*p* < 0.05). Intrathecal delivery of GSK872 inhibited the protein level of NF-κB p65, as shown in the GSK872 group (*p* < 0.05).

## Discussion

The current work mainly explores whether RIPK3 is a potential target for alleviating neuropathic pain after SCI. We are the first to find an increased expression of RIPK3 in the lumbar spinal dorsal horns of rats with thoracic SCI. Significantly, RIPK3 inhibitor GSK872 inhibited the expression of RIPK3 and alleviated mechanical hyperalgesia. Furthermore, we showed that GSK872 treatment reduced the expression of NLRP3 inflammasome, NF-κB, and proinflammatory factors, which indicated that RIPK3 inhibition effectively relieved neuroinflammation in the lumbar spinal dorsal horns.

Thoracic spinal cord contusion in male rats is the most commonly used model for studying SCI pain because this model matches clinical characteristics quite well in terms of trauma type and gender (Kramer et al., 2017). Mechanical stimulation and thermal stimulation were used to evaluate the pain behavior of neuropathic pain models. However, heat response in SCI models could represent exaggerated spinal reflexes (Shiao and Lee-Kubli, 2018). It has been reported that patients with SCI have a low probability of thermal hyperalgesia (Finnerup et al., 2014). In addition, van Gorp et al. (2014) found that rats that received severe thoracic contusion did not show low thermal withdrawal latency. In this study, we studied mechanical hyperalgesia of male rats with SCI and found that rats that received thoracic spinal cord contusion showed lower PWT than rats in the sham group, which indicated that the SCI neuropathic pain model was established.

Recently, RIPK3 has been well-studied in the neuropathic pain model caused by peripheral nerve injury. For instance, investigators reported that RIPK3 was highly expressed in the spinal cord, dorsal root ganglia, and hippocampus of the chronic constriction injury (CCI) model (Pu et al., 2018). He et al. (2021) further found that RIPK3 played an important role in CCI-induced neuropathic pain, and inhibition of RIPK3 ameliorated neuropathic pain *via* suppressing JNK signaling. We were curious about the role of RIPK3 in neuropathic pain after SCI. Our results indicated that rats with thoracic SCI exhibited high expressions of RIPK3, accompanied by remarkable mechanical allodynia from 7 to 21 days postoperation. It has been reported that the death signal ligand TNF-α activated RIPK3 and caused necrosis by binding to the death receptor (Vandenabeele et al., 2010). Our results showed that SCI led to increased TNF-α expression. Therefore, we speculated that TNF-α might be an important factor for causing the increased expression of RIPK3. Our observation also showed that RIPK3-specific inhibitor GSK872 could relieve allodynia and downregulate the expression of RIPK3 in the dorsal horns. Taken together, it suggested that the high expression of RIPK3 in the spinal dorsal horn could contribute to the development of neuropathic pain after SCI.

Inflammatory factors are the essential components of neuroinflammation. It has been demonstrated that proinflammatory factor is involved in neuropathic pain progress. Proinflammatory factors, such as IL-18, IL-1β, and TNF-α, are the famous pain mediators (Kawasaki et al., 2008; Pilat et al., 2016). The previous studies have indicated that increased expressions of TNF-α and IL-1β were associated with pain-related behaviors in a rat model of SCI (Detloff et al., 2008). The proinflammatory factor could induce SCI rat hyperalgesia *via* upregulating the excitability of superficial dorsal horn neurons (Fakhri et al., 2021). RIPK3 is a key kinase in regulating inflammatory factors (Yabal et al., 2014). Moreover, one research found that RIPK3 inhibition relieved neuropathic pain induced by peripheral nerve injury by decreasing proinflammatory factors' expressions (Fang et al., 2021). In this study, the proinflammatory factors were significantly increased in the spinal dorsal horns in SCI rats, accompanied by a reduction in mechanical pain threshold. Meanwhile, after intrathecal administration of GSK872, the proinflammatory factors such as IL-18, IL-1β, and TNF-α levels descended, with the relief of mechanical pain in rats with SCI. These results suggested that RIPK3 inhibition ameliorated mechanical allodynia possibly by reducing proinflammatory factors' expressions. However, the production mechanism of proinflammatory factors is not completely clear. We were curious about the upstream pathway of inflammatory factors.

Nod-like receptor family pyrin domain-containing protein 3 is a widely studied inflammasome that plays a vital role in the inflammatory immune response (Kelley et al., 2019). It has been reported that the dysregulation of the inflammasome is involved in a series of inflammatory diseases *via* promoting the secretion of inflammatory factors (Mangan et al., 2018). In the SCI model, we found that the expressions of NLRP3, caspase-1, and proinflammatory factors were increased in remote dorsal horns. In addition, immunofluorescence showed that NLRP3 was expressed in glial cells and neurons. These results indicated that NLRP3 inflammasome could contribute to the release of proinflammatory factors in glial and neurons, which also explain the potential mechanism of inflammatory factors released by the neurons and glial in remote dorsal horns. The growing evidence shows that NLRP3 may be a molecular target for neuropathic pain relief (Chen et al., 2021). For example, adenosine deaminase acting on RNA 3 (ADAR3) promoted pain relief in CCI rats by targeting NLRP3 inflammasome (Li et al., 2021). Our previous study also confirmed that caspase-1 inhibitor VX-765 could attenuate radicular pain *via* inhibiting NLRP3 inflammasome activation (Wang et al., 2020). In addition, D-β-hydroxybutyrate, one of the NLRP3 inflammasome inhibitors, effectively alleviated mechanical and thermal pain hypersensitivities in rats with SCI (Qian et al., 2017). The previous studies have confirmed that RIPK3 is a critical kinase regulating the NLRP3 inflammasome (Yabal et al., 2014). RIPK3 could contribute to NLRP3 inflammasome activation by producing ROS or potassium efflux (Moriwaki and Chan, 2017). The recent studies further suggested that RIPK3 is involved in the development of renal fibrosis (Shi et al., 2020), brain injury after subarachnoid hemorrhage (Zhou et al., 2017), and lung injury (Chen et al., 2018) by activating the NLRP3 inflammasome. Therefore, we speculated that the analgesic effect of RIPK3 inhibition might be connected with the reduction of proinflammatory cytokines by inhibiting the NLRP3 inflammasome. Intrathecal injection of GSK872 could significantly decrease the expressions of NLRP3, caspase-1, IL-1β, and IL-18 in the dorsal horns of model rats. These results further confirmed our conjecture.

The NF-κB p65, the main component of NF-κB pathways, is important in inflammatory and immune processes (Liu et al., 2017). When stimulated by external signals, NF-κB p65 separates from IκB and enters the nucleus to promote the transcription and expression of inflammatory genes related to pain (Mitchell and Carmody, 2018). NF-κB p65 has been shown to act as a contributor to neuropathic pain (Chu et al., 2020; Zhao et al., 2021). It has been reported that NF-κB inhibitor PDTC had a significant analgesic effect on CCI rats (Li et al., 2017). L-arginine, one of the NF-κB inhibitor, also relieved thermal pain hypersensitivity induced by SCI (Meng et al., 2017). An early investigation demonstrated that over-expression of RIPK3 could activate NF-κB signal (Kasof et al., 2000). The recent study further showed that RIPK3 inhibition could ameliorate osteoclastogenesis by regulating the NF-κB signaling pathway (Liang et al., 2020). Our experiments suggested that the expression of NF-κB p65 was significantly increased after SCI. Intrathecal injection of GSK872 could inhibit NF-κB p65 protein expression in SCI rat models. These results preliminarily indicated that anti-inflammatory and analgesic effects of RIPK3 inhibition are also related to the regulation of NF-κB pathways.

The previous studies mainly discussed the role of RIPK3-mediated necroptosis in secondary SCI, but few studies further explored the effects of regulating RIPK3 on neuroinflammation and pain. Sugaya et al. (2019) found that RIPK3 modulation prevented necroptosis of various nerve cells at the lesion site and favored neuroprotection. Dabrafenib treatment, one of the RIPK3 inhibitors, also promoted the recovery of motor function and sensory function after SCI. In this study, we studied the effects of RIPK3 modulation on pain and neuroinflammation. Our results suggested that the RIPK3 inhibition may mediate an anti-nociceptive effect by alleviating dorsal horn neuroinflammation. This study further emphasized the role of RIPK3 inhibition, which can not only alleviate necroptosis in the injured spinal cord and favor neuroprotection, but also reduce neuroinflammation and neuropathic pain after SCI.

Our study has some limitations, which need to be solved in the future research. This study preliminarily proved that RIPK3 inhibition could relieve mechanical allodynia of SCI rats. We did not further explore the effect of GSK872 dose change on mechanical allodynia. In this study, it was also not clear whether the inhibition of RIPK3 contributes to alleviating the neuroinflammation at the injured spinal cord and promoting the recovery of motor function.

## Conclusion

Our findings indicated that over-expressed RIPK3 developed mechanical allodynia in the SCI rat model. RIPK3 inhibition relieved mechanical allodynia possibly by suppressing NLRP3 inflammasome, NF-κB, and proinflammatory cytokines. Therefore, RIPK3 may be a potential target for treating neuropathic pain after SCI.

## Data Availability Statement

The original contributions presented in the study are included in the article/supplementary materials, further inquiries can be directed to the corresponding author.

## Ethics Statement

The animal study was reviewed and approved by Ethical Committee of the Shandong University.

## Author Contributions

SX and Z-xC carried out the major part of the study for making SCI model, detecting molecular indicators, the statistical analyses, and writing the manuscript. J-nW helped to conduct behavioral tests. Q-xZ performed the part of the western blot study. JH was in charge of part of the ELISA study. W-jY conducted the part of qRT-PCR. TS designed the study and revised the manuscript. All authors read and approved the final manuscript.

## Funding

This article was supported by the grants from the National Natural Science Foundation of China (grant no. 81772443 and 81972145) and Natural Science Foundation of Shandong Province (grant no. ZR2020MH283).

## Conflict of Interest

The authors declare that the research was conducted in the absence of any commercial or financial relationships that could be construed as a potential conflict of interest.

## Publisher's Note

All claims expressed in this article are solely those of the authors and do not necessarily represent those of their affiliated organizations, or those of the publisher, the editors and the reviewers. Any product that may be evaluated in this article, or claim that may be made by its manufacturer, is not guaranteed or endorsed by the publisher.
